# Exosomal miR-2276-5p in Plasma Is a Potential Diagnostic and Prognostic Biomarker in Glioma

**DOI:** 10.3389/fcell.2021.671202

**Published:** 2021-06-01

**Authors:** Jingxian Sun, Zhenying Sun, Ilgiz Gareev, Tao Yan, Xin Chen, Aamir Ahmad, Daming Zhang, Boxian Zhao, Ozal Beylerli, Guang Yang, Shiguang Zhao

**Affiliations:** ^1^Department of Neurosurgery, The First Affiliated Hospital of Harbin Medical University, Harbin, China; ^2^Institute of Brain Science, Harbin Medical University, Harbin, China; ^3^Central Research Laboratory, Bashkir State Medical University, Ufa, Russia; ^4^University of Alabama at Birmingham, Birmingham, AL, United States

**Keywords:** miRNA-2276-5p, circulating exosomes, glioma, RAB13, prognosis

## Abstract

**Introduction:**

Exosomal microRNAs (miRNAs) play an essential role in near and distant intercellular communication and are potential diagnostic and prognostic biomarkers for various cancers. This study focused on evaluation of exosomal miR-2276-5p in plasma as a diagnostic and prognostic biomarker for glioma.

**Methods:**

Plasma exosomes from 124 patients with glioma and 36 non-tumor controls were collected and subjected to quantitative real-time polymerase chain reaction (qRT-PCR) analysis for the exosomal miR-2276-5p expression. Bioinformatic analyses were performed to identify a gene target, and CGGA and TCGA databases were checked for evaluation of prognostic relevance.

**Results:**

The exosomal miR-2276-5p in glioma patients had a significantly decreased expression, compared with non-glioma patients (*p* < 0.01). Receiver operating characteristics (ROC) curve analyses were observed to regulate the diagnostic sensitivity and specificity of miR-2276-5p in glioma; the area under the curve (AUC) for miR-2276-5p was 0.8107. The lower expression of exosomal miR-2276-5p in patients with glioma correlated with poorer survival rates. RAB13 was identified as the target of miR-2276-5p which was high in glioma patients, especially those with higher tumor grades and correlated with poor survival.

**Conclusion:**

The circulating exosomal miR-2276-5p is significantly reduced in the plasma of glioma patients, and thus, it could be a potential biomarker for patients with glioma for diagnostic and/or prognostic purposes.

## Introduction

Glioma is a malignant disease with a high rate of mortality and morbidity. In 2018, there were 296,851 new glioma cases and 241,037 glioma patients’ deaths worldwide (Ferlay et al., 2019). According to the World Health Organization (WHO), glioma is classified as grades I–IV, with glioblastoma (GBM, grade IV) as the most malignant type (Ostrom et al., 2017; Capper et al., 2018). The primary treatment for GBM is surgical resection with radiotherapy and chemotherapy, and the median survival of patients is around 14–17 months (Reifenberger et al., 2017).

Exosomes, the small extracellular vesicles, are prevalent in biological fluids (Yoshimura et al., 2018) and can be invaluable biomarkers in cancer. The data for such role of exosomes in glioma are emerging (Huang et al., 2018). Exosomes contain nucleic acids and proteins, which play an essential role during glioma intercellular communication(s) (Qian et al., 2019). Exosomes’ concentration has been suggested as a biomarker in glioma, and it has been reported that Rabs, located on the specific organelle membranes, regulate each step of membrane transport. There are 60 Rabs or Rab-like proteins in cells (Hutagalung and Novick, 2011) that are all involved in the regulation of membrane transport. Rab proteins are also involved in the process of exosome formation (D’Souza-Schorey and Clancy, 2012), and their targeted knockdown can negatively impact the secretion of exosomes (Hoshino et al., 2013). Although miR-2276-5p is rarely reported in glioma, wherein it exists as an endogenous competitive RNA impacting glioma cells (Wang Z. et al., 2019), there has been no report on its possible evaluation as a potential biomarker in the exosomes for the diagnosis (or prognosis) of glioma patients. Furthermore, RAB13 has been reported as a novel biomarker in cancer (Chen et al., 2019a) with its expression correlating negatively with gastric cancer patients’ overall survival (OS) and progression-free survival (PFS). However, the role, if any, of RAB13 in glioma is unclear. Our study reports exosomal miR-2276-5p in GBM for the first time and further establishes RAB13 as the downstream of the miR-2276-5p. RAB13 could also predict the prognosis in glioma patients. This novel information should help design future studies to further explore the role of miR-2276-5p and its target RAB13 in glioma progression, with the aim to exploit this knowledge for development of targeted therapies against GBM.

## Materials and Methods

### Patients and Sample Preparation

The study was approved by the First Affiliated Hospital of the Harbin Medical University and implemented by the principles of the Helsinki Declaration. Patients signed informed consent forms before blood draw and surgery. We collected blood samples and clinical dates from 124 glioma patients, including three WHO grade I patients, 30 grade II patients, 34 grade III patients, and 57 grade IV patients who underwent treatment in the Department of Neurosurgery at the First Affiliated Hospital of Harbin Medical University in China between July 2015 and July 2017. The patients were diagnosed with glioma by postoperative pathological section according to the WHO criteria, and pathological diagnosis was performed by two independent pathologists. Non-glioma patients who had no medical history of another cancer were recruited into this study as controls (*n* = 36), matched by sex and age with the glioma group. Clinical characteristics of the glioma patients are summarized in Table 1. All plasma samples for glioma and non-tumor patients were collected in EDTA-K3 tubes before the start of any treatment. After the first centrifugation for 10 min at 3,000 × *g*, the supernatants were carefully moved to a new tube and snap frozen at −80°C to isolate the exosomes.

**TABLE 1:** The Baseline Characteristics of Glioma Patients.

Characteristics	*n* = 124
Age, y	50.7 ± 14.1
**Grade**	
I	3 (2.4)
II	30 (24.2)
III	34 (27.4)
IV	57 (46.0)
**Gender**	
Male	54 (43.5)
Female	70 (56.5)
Survival Time (Month)	13.18 ± 11.30
**Location of Glioma**	
Frontal	66 (53.2)
Temporal	40 (32.3)
Parietal	10 (8.1)
Occipital	5 (4.0)
Infratentorial	3 (2.4)
**Extent of Surgery**	
Complete resection	85 (78.5)
Biopsy or partial resection	39 (31.5)

### Cell Lines and Transfections

Human glioblastoma cell lines (LN229 and U87) were obtained from China Infrastructure of Cell Line Resource (National Science and Technology Infrastructure, NSTI). They were maintained in DMEM medium, and 10% fetal bovine serum (Biological Industries, Israel) was added to the medium. The miR-2276-5p mimics and negative control (NC) were purchased from the General Biosystems (Anhui, China), and Lipofectamine 2000 was purchased from Invitrogen (United States). Before transfection, the glioma cells LN229 and U87 were cultured in six-well plates at a density of 6 × 10^4^ cells per well, and transfections were performed according to the manufacturer’s instructions.

### Exosome Isolation From Plasma

Five hundred microliters of plasma samples was centrifuged at 4°C at 300 × *g* for 10 min, 1,000 × *g* for 10 min, and 10,000 × *g* for 30 min to remove cells and debris. Then, supernatants were subjected to ultracentrifugation at 100,000 × *g* for 70 min. Next, we discarded the supernatants and used phosphate-buffered saline (PBS) to wash exosomes. Finally, the exosomes were pelleted by ultracentrifugation at 100,000 × *g* for 70 min again and resuspended in 100 μl of PBS for RNA isolation and follow-up use.

### Transmission Electron Microscope

The use of transmission electron microscopy was consistent with that of previous reports, and the experimental procedures used in previous experiments were used (Wang et al., 2018). The exosome resuspension was loaded onto a carbon-coated 300 mesh copper grid and dried at room temperature for 5 min. Then, the grid was dyed with the 2% phosphotungstic acid at room temperature for 10 min, and the morphology of exosomes was checked using an electron microscope (JEM-1220, JEOL Ltd., Japan).

### Protein Extraction and Western Blot

Extraction of proteins and Western blot (WB) analysis was performed as reported earlier (Wang et al., 2018). CD63 antibody (25682-1-AP, 1:1,000), CD9 antibody (20597-1-AP, 1:1,000), and GAPDH (60004-1-LG, 1:5,000) were purchased from Proteintech. RAB13 antibody (DF9813, 1:1,000) was purchased from Affinity Biosciences. Polyclonal goat anti-rabbit antibody (SA00001-2, Proteintech) was used as the secondary antibody, and the WB detection system (Gene Sys) was used for generation of data.

### MTT

Transfected glioma cells LN229 and U87 were seeded in 96-well plates at a cell density of 1 × 10^4^ cells per well, and 20 μl of MTT was added at 24, 48, and 72 h. The test conditions were similar to our previous studies (Zhong et al., 2019).

### RNA Extraction and Quantitative Real-Time Polymerase Chain Reaction

Total exosome RNA was isolated from 100 μl of exosome samples using Trizol and stored in a –80°C freezer until use. U6 was used as an endogenous control miRNA that is stably expressed in exosomes. qRT-PCR was performed as described earlier (Gareev et al., 2019). The relative expression of miR-2276-5p was calculated using the equation 2^–ΔCt^, in which ΔCt = Ct miR-2276-5p-Ct U6. The primer sequences used in this study are reported in Supplementary Table 1.

### Bioinformatic Tools

We consulted GEPIA^1^, GEO^2^, and GlioVis^3^ to check the expression level of RAB13 in the glioma patients and the outcome survival of RAB13 expression. TargetScan^4^, mirDIP^5^, and mirtargetbase^6^ were checked to calculate the gene targets of miR-2276-5p.

### Statistical Analyses

Statistical analyses were analyzed by SPSS 13.0. Data were expressed as mean ± SEM. Kaplan–Meier analysis was used to generate and analyze survival time data. The univariate Cox proportional hazards regression was performed on SPSS 13.0. The differences were considered statistically significant at *p* < 0.05.

## Results

### Identification of Plasma Exosomes

**FIGURE 1:**
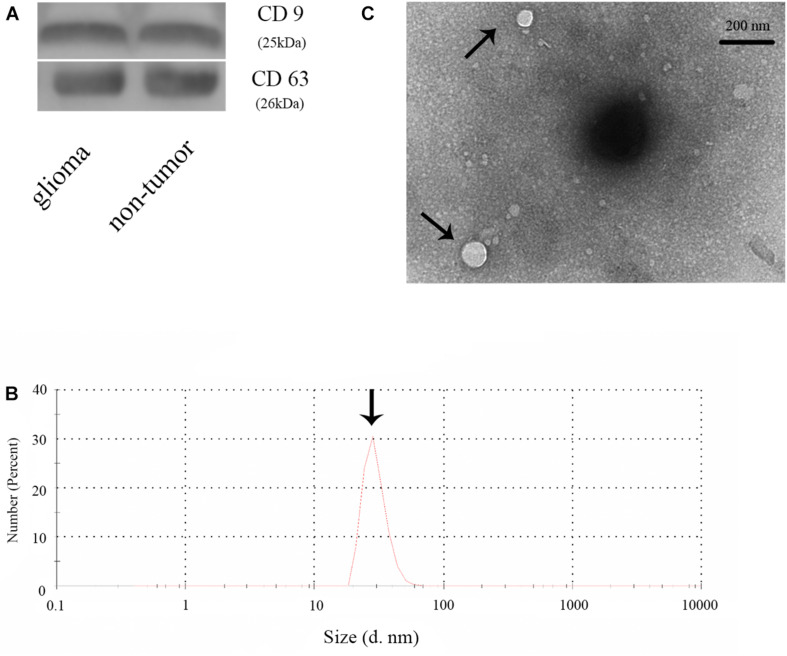
Identification of plasma exosomes. **(A)** The expression of CD9 and CD63 in plasma exosomes in glioma and non-glioma patients. **(B)** Determination of the exosome diameter by Nano-Sight. **(C)** Transmission electron microscope image of the plasma exosomes.

To confirm the extraction of exosomes from the plasma of glioma and control patients, the markers of exosomes (CD63 and CD9) were evaluated by WB analysis. The result showed CD63 and CD9 expression in the samples, thus, establishing the presence of exosomes (Figure 1A). Then, we checked the diameter of exosomes using Nano-Sight (Figure 1B) and found the size of exosomes to be distributed below 100 nm. We further examined the morphology of exosomes and verified our findings using transmission electron microscope (Figure 1C). Thus, we concluded that we had successfully extracted exosomes from plasma samples.

### Exosomal miR-2276-5p Is Unconventionally Expressing in Glioma Patients and Could Be a Potential Diagnostic Biomarker in Glioma

**FIGURE 2:**
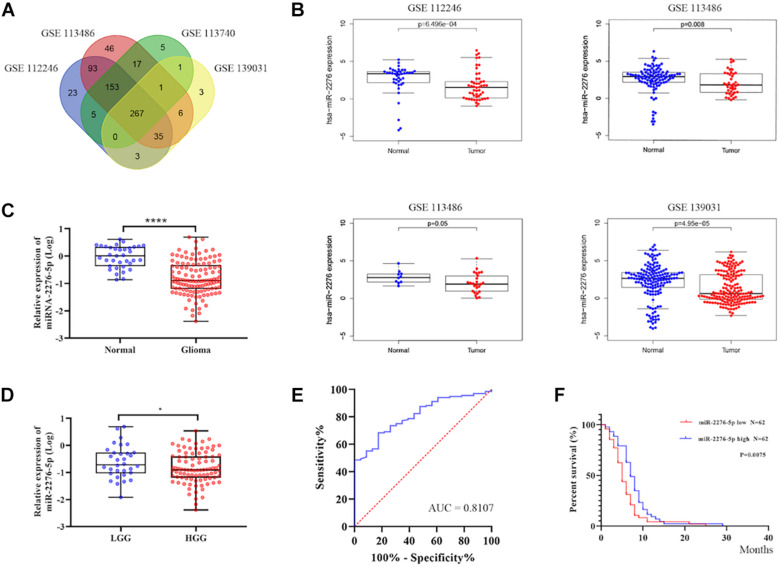
Exosomal miR-2276-5p could be a potential diagnostic and prognostic biomarker in glioma. **(A,B)** The relative expression of miR-2276-5p in glioma patients’ plasma in GSE139031, GSE113740, GSE113486, and GSE112246. **(C)** The relative expression of miR-2276-5p in glioma patients’ plasma exosomes. **(D)** The relative expression of miR-2276-5p in LGG and HGG patients’ plasma exosomes. **(E)** The receiver operating characteristics analysis curve of the miR-2276-5p and the AUC was 0.8107. **(F)** Correlation of expression level of exosomal miR-2276-5p with the overall survival rate of glioma patients. *p < 0.05, ****p < 0.0001.

As shown in Figures 2A,B, GSE139031, GSE113740, GSE113486, and GSE112246 reported a significantly reduced expression of miR-2276 in glioma. To further understand whether miR-2276-5p is also differentially expressed in the plasma exosomes of glioma patients, we checked miR-2276-5p in the exosomes isolated from the plasma of glioma patients and controls. The expression of exosomal miR-2276-5p was found to be differential between glioma and non-glioma patients’ plasma (Figure 2C). The results showed that plasma exosomal miR-2276-5p in glioma is significantly lower, compared with the non-glioma controls (Figure 2C). Another interesting revelation was that the exosomal miR-2276-5p expression levels were lower in patients with high-grade glioma (HGG, including grade III and grade IV) than in patients with low-grade glioma (LGG, including grade I and grade II) (Figure 2D). The result of univariate logistic regression analysis of factors associated with a risk factor for glioma (Table 2). Then a receiver operating characteristics (ROC) analysis curve was used to analyze the predictive diagnostic capability of exosomal miR-2276-5p for glioma patients. Admission exosomal miR-2276-5p had a good area under curve with an AUC value of 0.8107 (Figure 2E). These results suggested that the exosomal miR-2276-5p expression levels are markedly decreased in glioma patients and further correlate negatively with the tumor grade.

**TABLE 2:** The result of univariate logistic regression analysis in glioma patients.

	Univariable analysis OR	95% CI	*P*-value
Relative expression of miR-2276-5p	0.334	0.204-0.548	<0.01

### Exosomal miR-2276-5p in Plasma Could Be a Potential Prognostic Biomarker in Glioma

We further explored whether there existed a relationship between these differences in expression and the survival outcome of the patients. The relative expression of exosomal miR-2276-5p in patients was divided into a high-expression group and a low-expression group, and our analysis showed that glioma patients in the low-expression group of exosomal miR-2276-5p had lower overall survival (Figure 2F). We used the univariate and multivariable Cox proportional hazards regression to confirm our findings (Table 3). Based on these findings, we suggest that plasma exosomal miR-2276-5p expression can be used as an independent factor to predict the survival of patients with glioma.

**TABLE 3:** The univariate and multivariable Cox proportional hazards regression in glioma patients.

	Univariate		Multivariable	
	Hazard ratio (95% CI)	*P*-value	Hazard ratio (95% CI)	*P*-value
Relative expression of miR-2276-5p	0.619 (0.408–0.940)	0.024	0.573 (0.354–0.926)	0.023
Grade	4.379 (3.066–6.252)	<0.001	3.987 (2.738–5.806)	<0.001
Age	1.047 (1.031–1.063)	<0.001	1.021 (1.004–1.037)	0.014
Gender (VS Female)	0.791 (0.523–1.197)	0.268	0.895 (0.584–1.374)	0.613
Total cut	0.813 (0.530–1.249)	0.345	0.907 (0.579–1.420)	0.669

### RAB13 May Be the Target Gene of the Plasma Exosomal miR-2276-5p Which Also Predicts the Glioma Patients’ Survival

**FIGURE 3:**
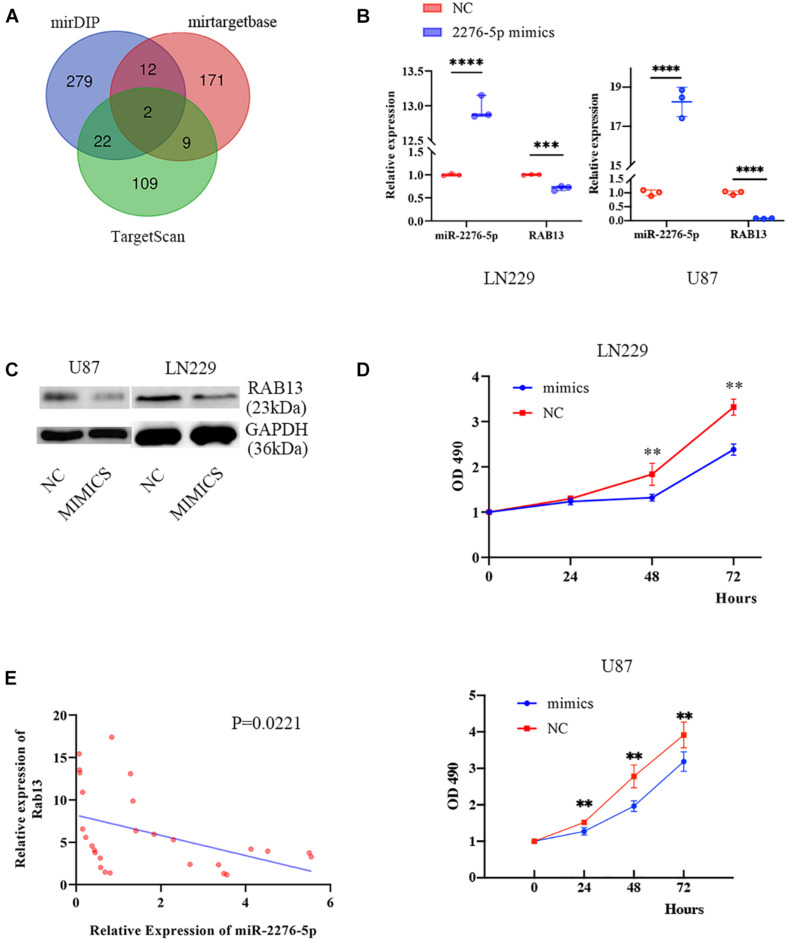
RAB13 may be the target gene of miR-2276-5p. **(A)** The predicted result of target genes of miR-2276-5p, as determined by consultation with mirDIP, mirtargetbase, and TargetScan. **(B)** The relative expression of miR-2276-5p and RAB13 mRNA in LN229 and U87 glioma cell lines transfected with miR-2276-5p or the negative control (NC) mimics. **(C)** Western blotting results showing the expression levels of RAB13 protein in miR-2276-5p/NC- transfected LN229 and U87 glioma cell lines. **(D)** The cell proliferation state of miR-2276-5p/NC-transfected LN229 and U87 glioma cells, as determined by MTT. **(E)** The correlation of expression level of miR-2276-5p with the expression levels of RAB13 in glioma tissues. ^∗∗^*p* < 0.01; ^∗∗∗^*p* < 0.001; ^****^p < 0.0001.

**FIGURE 4:**
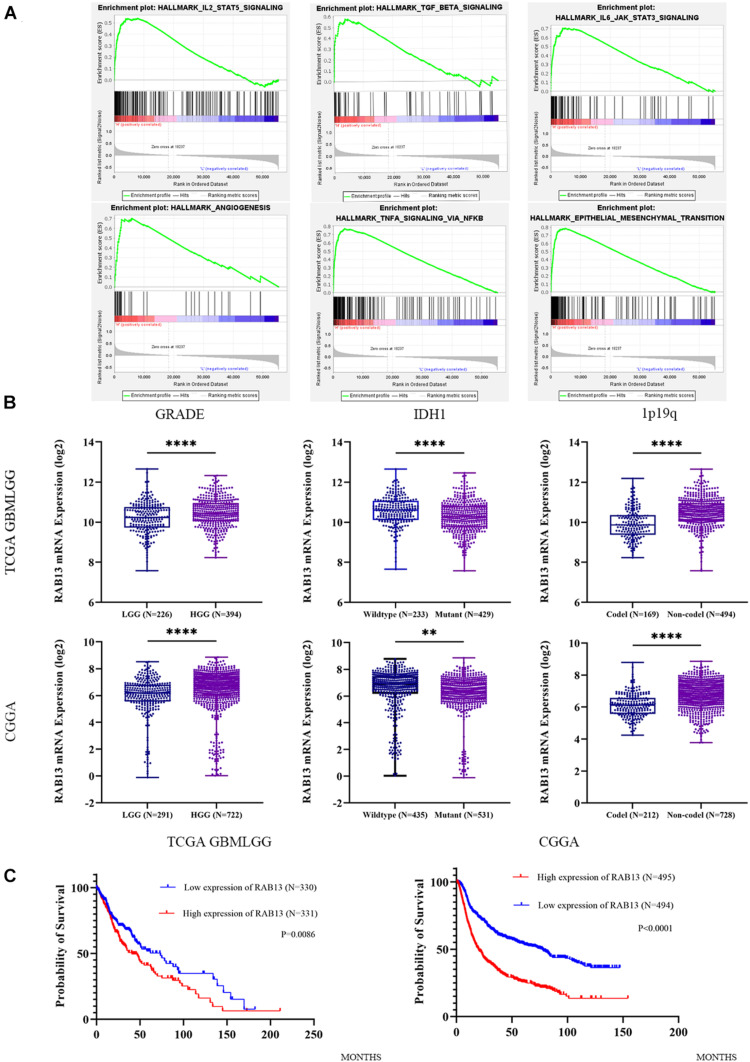
The expression of RAB13 in the glioma database is correlated with the clinical indicators of the patient. **(A)** The enrichment result of RAB13 by GSEA analysis, using the TCGA database. **(B)** The relative expression of RAB13 mRNA in the TCGA and CGGA databases by grouping according to the grade, IDH1, and 1p19q status. **(C)** Correlation of expression level of RAB13 with the overall survival rate of glioma patients in the TCGA and CGGA databases. ^∗∗^*p* < 0.01; ^****^p < 0.0001.

To better understand which genes are the targets of miR-2276-5p in glioma, we used mirDIP, mirtargetbase, and TargetScan to predict the target genes and found that RAB13 and PLEKHG48 might be the target of miR-2276-5p (Figure 3A). However, PLEKHG48 had no difference in expression in glioma patients, and it was not necessarily related to the prognosis of patients in the TCGA database (Supplementary Figure 1). We transfected miR-2276-5p mimics and negative control (NC) miRNA into the LN229 and U87 human glioma cell lines and found that when miR-2276-5p was ectopically highly expressed in LN229 and U87 glioma cells, the expression of RAB13 mRNA in cells was decreased, as evaluated by RT-PCR (Figure 3B). We further confirmed these results with Western blotting (Figure 3C); the protein expression of RAB13 also decreased in both the glioma cell lines. Furthermore, the survival of miR-2276-5p-transfected LN229 as well as U87 cells was worse than in the NC group (Figure 3D). Moreover, we evaluated the tumor tissues obtained from our hospital and obtained the results that showed that the expression of RAB13 mRNA was negatively correlated with miR-2276-5p (Figure 3E). Based on these results, we speculate that RAB13 may be a target gene for miR-2276-5p. We used GSEA to analyze the TCGA-related data and analyzed the cell signaling pathways related to RAB13. Our analysis revealed that RAB13 was enriched in IL2-STAT5 signaling pathway, TGF-β signaling pathway, IL6-JAK-STAT3 signaling pathway, angiogenesis signaling pathway, TNF-α signaling pathway, and epithelial–mesenchymal transition (Figure 4A). Furthermore, in order to clarify that RAB13 was correlated with the overall survival of patients, the expression level of RAB13 was checked in the CGGA and TCGA databases. The expression level of RAB13 in HGG patients was significantly higher than in LGG patients (Figure 4B). Next, we classified the patients in the CGGA and TCGA database via IDH1 and 1p19q status; RAB13 was highly expressed in IDH1 wild-type and 1p19q non-codel patients (Figure 4B). We verified using the GlioVis database that the prognosis of patients with high RAB13 expression was significantly worse than that of patients with low RAB13 expression in both the CGGA and TCGA patients (Figure 4C). Thus, we conclude that RAB13 is a genuine target of miR-2276-5p, which is differentially expressed in glioma patients, relative to the controls, as well as in LGG vs. HGG patients, making it a putative indicator to predict the prognosis of glioma patients.

## Discussion

In our current study, we found that exosomal miR-2276-5p in plasma may be a diagnostic biomarker in glioma patients and that this miRNA is associated with a poor prognosis in patients with lower expression levels. Moreover, we conclude that miR-2276-5p is likely to target RAB13 and inhibit glioma cell growth. In published literature, miR-2276-5p has been reported dysregulated in breast cancer (Torkashvand et al., 2016) and colorectal cancer (Chen et al., 2019a), and the researchers used the predictive tools to suggest a relationship between miR-2276 and PIWIL2. In another report, there was a 4.5-fold high expression of miR-2276 in colorectal cancer cells that were silenced for STAT3. STAT3 is a well characterized tumor promotor in glioma (Zhang et al., 2014; Lv et al., 2017; Almiron Bonnin et al., 2018; Man et al., 2018). It can promote glioma cell proliferation and glioma stem-like cell self-renewal. Thus, suppression of miR-2276 by oncogenic STAT3 establishes it as a tumor suppressive miRNA in gliomas.

Exosomes are 40–150 nm small extracellular vesicles, which are involved in the regulation of cellular microenvironment. They carry cargo such as RNA (including mRNAs, microRNAs, and non-coding RNAs), DNA, protein, and lipids to facilitate communications between cancer cells (Jeppesen et al., 2019; Khan et al., 2020). During the past several years, there have been many reports on miRNAs in glioma. Not only the miRNAs, such as, miR-21 (Masoudi et al., 2018), miR-454-3p (Van Meir et al., 2010), and miR-9 (Chen et al., 2019b), but even reports on non-coding RNAs, such as long non-coding RNA HOTAIR (Tan et al., 2018), have suggested a possible role of non-coding RNAs as biomarkers in glioma and asserted that they were a risk factor for the prognosis in glioma patients. This fits wells with the increasing realization of a role of non-coding RNAs in the pathogenesis of human cancers (Ahmad, 2016; Kim, 2019). It has been reported that miR-26a promotes tumor angiogenesis; it activates PI3K/Akt signaling pathway by targeting PTEN, thus, promoting glioma cells’ proliferation (Wang Z.F. et al., 2019). Previous studies have found that the concentration of extracellular vesicles can be a factor in predicting the prognosis of patients with glioma (Osti et al., 2019), but it was not clear as to why the glioma patients had high concentration of extracellular vesicles. According to our findings, as reported here, we suspect that the low expression of miR-2276-5p would promote the expression of RAB13, which in turn can positively regulate the secretion of transport vesicles between glioma cells, and finally lead to the secretion of a large number of extracellular vesicles. Our work also links miR-2276-5p to glioma through exosomes for the first time, and we found a relationship between the expression of this exosomal miRNA and the diagnosis and prognosis of glioma patients.

RAB13 regulates transport and location function proteins. These include integrin transport during cell proliferation and migration, GLUT4 and VEGFR during angiogenesis (Munn et al., 1998; Sun et al., 2010; Nishikimi et al., 2014). RAB13 regulates the proliferation of cancer cells and tumor growth *in vivo*. Therefore, RAB13 is involved in the regulation of several key determinants of cancer cells aggressiveness (Ioannou et al., 2015). We found, through bioinformatics analysis, that RAB13 is mainly enriched in the JAK/STAT3 signaling pathway. The JAK/STAT3 signaling pathway has previously been shown to be critical in glioma. By inhibiting the JAK-STAT3 signaling pathway, the growth and invasion of glioma cells could be significantly inhibited, primarily through the inhibition of cell cycle in the G0/G1 phase (Lu et al., 2017). We also found that RAB13 is significantly enriched in the signaling pathway of angiogenesis. Angiogenesis is a significant feature of gliomas, and inhibition of the formation of blood vessels has direct effect on the treatment and prognosis of gliomas (Shi et al., 2014). Studies have also found that activation of STAT3 could induce tumor angiogenesis (Yu and Jove, 2004). Therefore, we infer that the low expression of miR-2276-5p leads to the differential expression of RAB13, and RAB13 may promote glioma angiogenesis through the JAK/STAT3 signaling pathway, leading to tumor proliferation and poor prognosis. Clearly, more experimental proof is needed to verify this and should be the focus of future studies.

In conclusion, we report that miR-2276-5p is reduced in the plasma-derived exosomes of patients with glioma, and its low expression associates with poor prognosis of patients. Additionally, we found, using bioinformatic tools, that RAB13 is differentially expressed in patients with glioma, and predicted and verified it as a target of miR-2276-5p. Our findings suggest that exosomal miR-2276-5p in plasma is a potential diagnostic and prognostic biomarker for glioma.

## Data Availability Statement

The original contributions presented in the study are included in the article/Supplementary Material, further inquiries can be directed to the corresponding author/s.

## Ethics Statement

The studies involving human participants were reviewed and approved by the First Affiliated Hospital of the Harbin Medical University. The patients/participants provided their written informed consent to participate in this study.

## Author Contributions

JS, ZS, IG, and TY performed the experiments. JS, ZS, IG, OB, DZ, BZ, and GY analyzed the data. GY and SZ provided the funds. OB, AA, GY, and SZ conceptualized the study. OB, GY, and SZ drafted the manuscript. All authors read and approved the manuscript.

## Conflict of Interest

The authors declare that the research was conducted in the absence of any commercial or financial relationships that could be construed as a potential conflict of interest.

## Footnotes

1 http://gepia.cancer-pku.cn

2 https://www.ncbi.nlm.nih.gov/geo

3 http://gliovis.bioinfo.cnio.es/

4 http://www.targetscan.org/vert_72

5 http://ophid.utoronto.ca/mirDIP/

6 http://mirdb.org/

## Supplementary Material

Supplementary Figure 1 The relationship between the expression of PLEKHG48 and the diagnosis and prognosis of patients with glioma.

Supplementary Table 1 The detailed sequence of miRNAs.
