# Supplementary figures and images for: Exosomal miR-2276-5p in Plasma Is a Potential Diagnostic and Prognostic Biomarker in Glioma
Source: Front Cell Dev Biol. 2021 Jun 1;9:671202. doi: 10.3389/fcell.2021.671202 (PMC8204016; doi:10.3389/fcell.2021.671202)

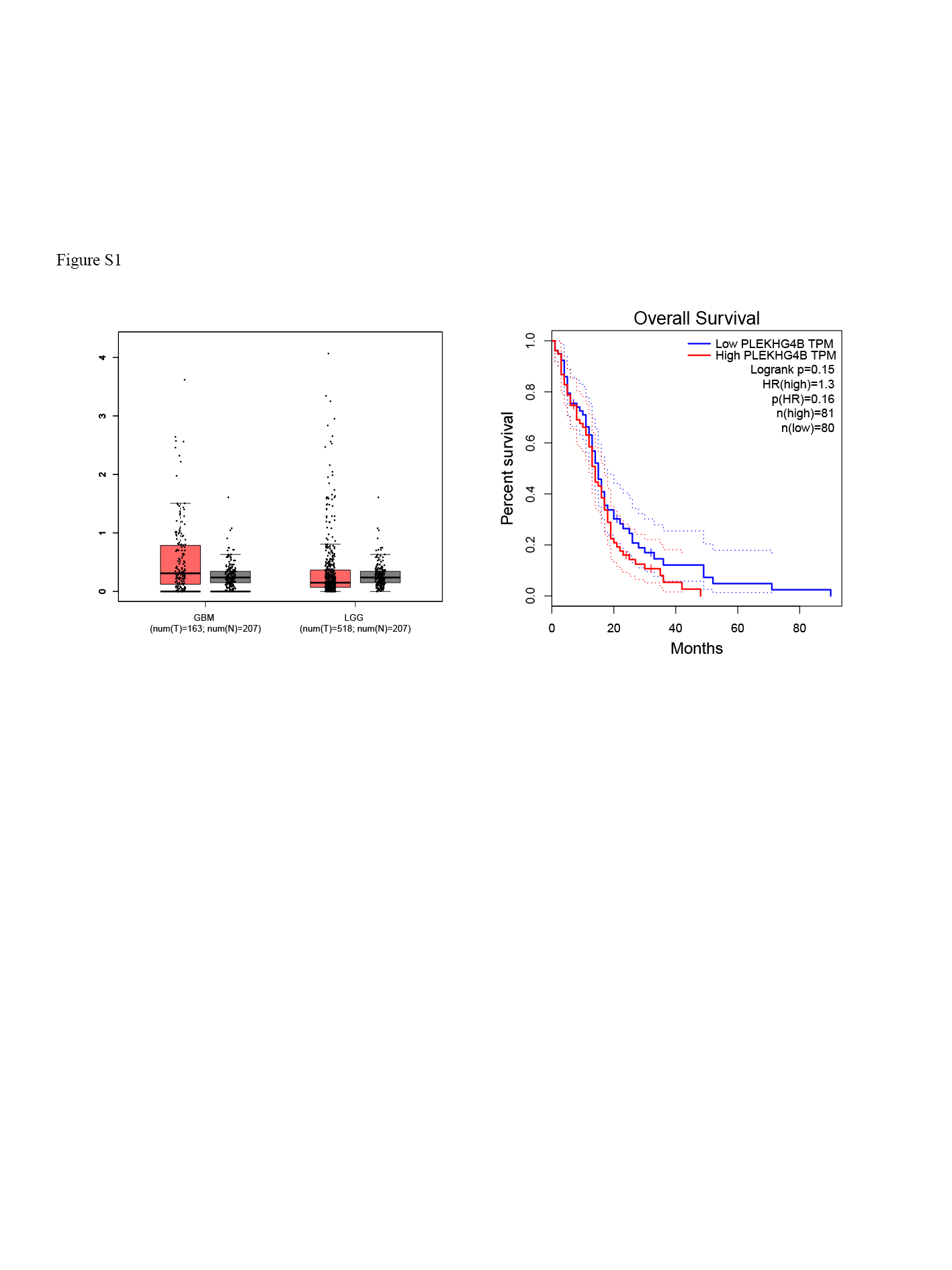

Supplement: Supplementary Figure 1 — The relationship between the expression of PLEKHG48 and the diagnosis and prognosis of patients with glioma. [file Image_1.TIF]
