# Supplementary material for: Exosomal miR-2276-5p in Plasma Is a Potential Diagnostic and Prognostic Biomarker in Glioma
Source: Front Cell Dev Biol. 2021 Jun 1;9:671202. doi: 10.3389/fcell.2021.671202 (PMC8204016; doi:10.3389/fcell.2021.671202)
Supplement: Supplementary Table 1 — The detailed sequence of miRNAs. [file Table_1.doc]

|  | Primer Sequence (5’-3’) |
| --- | --- |
| miRNA-2276-5p | RT: GTCGTATCCAGTGCAGGGTCCGAGGTATTCGCACTGGATACGACCCCAAC  forward: GCGGCCCTCTGTCACCTTG  reverse: ATCCAGTGCAGGGTCCGAGG |
| Rab13 | forward: AAGGAGCAGGCCGATAAGTT  reverse: GTTGCCGTTTCCTGATCTCC |
| GAPDH | forward: TGATGACCCTTTTGGCTCCC;  reverse: GAAGCTTGTCATCAATGGAAAT |
| miRNA-2276-5p mimics | forward: GCCCUCUGUCACCUUGCAGACG |
|  | reverse: CGUCUGCAAGGUGACAGAGGGC |
| NC | forward: UUCUUCGAACGUGUCACGUTT |
|  | reverse: ACGUGACACGUUCGGAGAATT |

**Supplementary Table 1. The detailed sequence of miRNAs.**
